# The Rubisco small subunits in the green algal genus *Chloromonas* provide insights into evolutionary loss of the eukaryotic carbon-concentrating organelle, the pyrenoid

**DOI:** 10.1186/s12862-020-01733-1

**Published:** 2021-01-25

**Authors:** Ryo Matsuzaki, Shigekatsu Suzuki, Haruyo Yamaguchi, Masanobu Kawachi, Yu Kanesaki, Hirofumi Yoshikawa, Toshiyuki Mori, Hisayoshi Nozaki

**Affiliations:** 1 Center for Environmental Biology and Ecosystem Studies, National Institute for Environmental Studies, Onogawa, Tsukuba, Ibaraki 305-8506 Japan; 2 Faculty of Life and Environmental Sciences, University of Tsukuba, Tsukuba, Ibaraki 305-8572 Japan; 3 Research Institute of Green Science and Technology, Shizuoka University, Shizuoka, 422-8529 Japan; 4 NODAI Genome Research Center, Tokyo University of Agriculture, Setagaya-ku, Tokyo 156-8502 Japan; 5 Department of Bioscience, Tokyo University of Agriculture, Setagaya-ku, Tokyo 156-8502 Japan; 6 Department of Tropical Medicine and Parasitology, Juntendo University, 2-1-1 Hongo, Bunkyo-ku, Tokyo 113-8421 Japan; 7 Department of Biological Sciences, Graduate School of Science, The University of Tokyo, 7-3-1 Hongo, Bunkyo-ku, Tokyo 113-0033 Japan

**Keywords:** *Chloromonas*, Evolution, Green algae, Hydrophobicity of RBCS helices, Pyrenoid, Pyrenoid loss, Rubisco small subunit (RBCS)

## Abstract

**Background:**

Pyrenoids are protein microcompartments composed mainly of Rubisco that are localized in the chloroplasts of many photosynthetic organisms. Pyrenoids contribute to the CO_2_-concentrating mechanism. This organelle has been lost many times during algal/plant evolution, including with the origin of land plants. The molecular basis of the evolutionary loss of pyrenoids is a major topic in evolutionary biology. Recently, it was hypothesized that pyrenoid formation is controlled by the hydrophobicity of the two helices on the surface of the Rubisco small subunit (RBCS), but the relationship between hydrophobicity and pyrenoid loss during the evolution of closely related algal/plant lineages has not been examined. Here, we focused on, the *Reticulata* group of the unicellular green algal genus *Chloromonas*, within which pyrenoids are present in some species, although they are absent in the closely related species.

**Results:**

Based on de novo transcriptome analysis and Sanger sequencing of cloned reverse transcription-polymerase chain reaction products, *rbcS* sequences were determined from 11 strains of two pyrenoid-lacking and three pyrenoid-containing species of the *Reticulata* group. We found that the hydrophobicity of the RBCS helices was roughly correlated with the presence or absence of pyrenoids within the *Reticulata* group and that a decrease in the hydrophobicity of the RBCS helices may have primarily caused pyrenoid loss during the evolution of this group.

**Conclusions:**

Although we suggest that the observed correlation may only exist for the *Reticulata* group, this is still an interesting study that provides novel insight into a potential mechanism determining initial evolutionary steps of gain and loss of the pyrenoid.

## Background

A pyrenoid is a protein body that is often surrounded by a starch sheath and is located in the chloroplast stroma of most eukaryotic algae and some hornwort species. The pyrenoid contributes to the CO_2_-concentrating mechanism [1–4]. This organelle consists mainly of ribulose-1,5-bisphosphate carboxylase/oxygenase (Rubisco), a key photosynthetic enzyme composed of eight large and eight small subunits [5]. Molecular phylogenetic analyses have suggested that the pyrenoid has been lost many times independently during the evolution of photosynthetic organisms [4, 6, 7], including during the origin of land plants [7]. Although recent papers have demonstrated that some Rubisco-binding proteins directly regulate pyrenoid morphology [8, 9], the molecular basis of the evolutionary loss of pyrenoids is largely unknown.

Based on transformational experiments of the unicellular pyrenoid-containing green alga *Chlamydomonas reinhardtii*, Meyer et al. found that absence of two *C. reinhardtii* helices of the Rubisco small subunit (RBCS) results in no pyrenoid formation even though the spinach RBCS helices are present in the Rubisco [10]. They suggested that hydrophobic interactions between Rubisco molecules within the pyrenoid via two helices on the surface of RBCS are correlated with pyrenoid formation [10]. A substantial amount of work has subsequently highlighted that the interaction of the linker protein “Essential Pyrenoid Component 1 (EPYC1)” with RBCS, specifically the two helices, is critical for pyrenoid formation [8, 11, 12]. Recently, Goudet et al. [13] examined RBCS sequences across chlorophyte and streptophyte green algae and concluded that specific residues in the RBCS helices [10] were not sufficient to explain the pyrenoid occurrence across the entire green algal phylum. However, the relationship between the hydrophobicity of the two RBCS helices and presence or absence of pyrenoids within a closely related lineage has not been studied.

The unicellular green algal genus *Chloromonas* belongs to *Chloromonadinia*, a strongly supported primary clade [14] in the order Chlamydomonadales (= Volvocales), and includes both pyrenoid-containing and -lacking species [6, 15, 16]. Within this genus, the *Reticulata* group is a small clade that includes at least three pyrenoid-containing species and two pyrenoid-lacking species; based on the phylogenetic tree constructed and distribution of presence or absence of pyrenoids in operational taxonomic units, it has been suggested that pyrenoids have been lost twice during the evolution of this group [17]. The CO_2_-concentrating mechanism, chloroplast ultrastructure, and Rubisco distribution in the chloroplast have been studied extensively in several strains and species belonging to the *Reticulata* group [6, 18]. Many amino acid substitutions were found in the Rubisco large subunit (RBCL) in the *Reticulata* group or the genus *Chloromonas* and a possible relationship between RBCL substitutions and loss of pyrenoids was suggested [15]. However, the nuclear *rbcS* genes in this group have not been studied.

Here, we determined the *rbcS* sequences of 11 strains of five species in the *Reticulata* group [17] using de novo transcriptome analysis and Sanger sequencing of cloned reverse transcription-polymerase chain reaction (RT-PCR) products. We found that the hydrophobicity of the RBCS helices was correlated with the presence or absence of the pyrenoid within the *Reticulata* group.

## Results

### Phylogeny of 11 *Chloromonas *strains of the *Reticulata* group

**Fig. 1:**
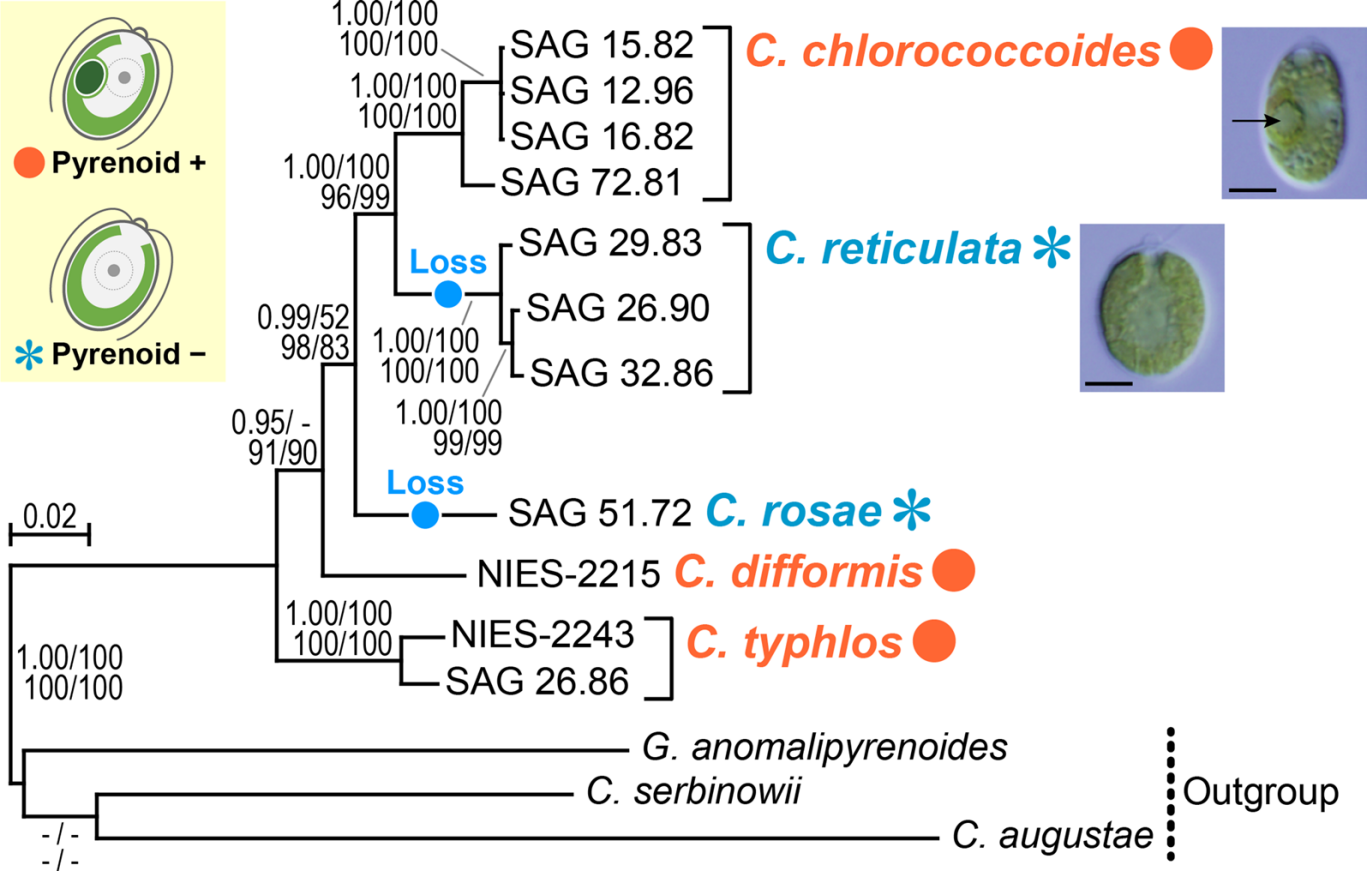
Bayesian phylogenetic tree of the *Reticulata* group of the genus *Chloromonas* based on the combined 7,109-bp data matrix for nuclear 18S and 28S ribosomal DNA, *atpB*, *psaA*, *psaB*, and ITS-2 sequences, showing evolution of pyrenoids. Numbers shown in top left, top right, bottom left, and bottom right exhibit posterior probabilities (0.95 or more) from Bayesian inference and bootstrap values (50% or more) from maximum likelihood, maximum parsimony, and neighbor-joining analyses, respectively. Presence (+) or absence (−) of pyrenoids in the *Reticulata* group is based on the previous light and electron microscopic studies [16–18]. Pyrenoid loss was deduced by using Mesquite V3.6 [47]. In light microscopic photographs, vegetative cells of *C. chlorococcoides* strain SAG 15.82 and *C. reticulata* strain SAG 29.83 are shown, bars represent 5 μm and an arrow indicates a pyrenoid

The sister relationship between *C. chlorococcoides* and *C. reticulata* was more robustly resolved [with 1.00 posterior probability in Bayesian inference (BI) and 96–100% bootstrap values [19] in three other phylogenetic methods] than in the previous study [17] (Fig. 1). Although the bootstrap value by maximum likelihood (ML) method was low (52%), *C. rosae* was sister to the clade composed of *C. chlorococcoides* and *C. reticulata* [with 0.99 posterior probability in BI and 83–98% bootstrap values in maximum parsimony (MP) and neighbor-joining (NJ) methods]. It was suggested that pyrenoids have been lost twice during the evolution of this group (Fig. 1).

Obvious contradictions of the single markers in phylogenetic positions of *C. rosae*, *C. difformis* and *C. typhlos* were recognized, but only supported with low statistical supports in 28S ribosomal DNA- and P700 chlorophyll *a*-apoprotein A1 gene (*psaA*)*-*based trees (Additional file 1: Fig. S1). Only ML analysis (with 58% bootstrap value) resolved sister relationship between *C. difformis* and *C. typhlos* in the 28S ribosomal DNA tree. The *psaA* tree suggested sister relationship between *C. difformis* and the other four *Chloromonas* species with 0.95 posterior probability in Bayesian inference and 57% bootstrap value in NJ method (Additional file 1: Fig. S1). In contrast, the phylogenetic positions of *C. rosae*, *C. difformis* and *C. typhlos* supported with high bootstrap values (83–98%) in NJ and MP analyses of the concatenated data set (Fig. 1) were also resolved in the ITS-2 tree with 90–96% bootstrap values in NJ and MP methods (Additional file 1: Fig. S1). In addition, the previous phylogenetic analysis based on the combined data set lacking ITS-2 sequences resolves the most basal position of *C. typhlos* with 51–71% bootstrap values in ML, NJ and MP calculations, but does not demonstrate 50% or more bootstrap values or 0.95 or more posterior probability in the four phylogenetic methods for supporting the sister relationship between *C. rosae* and *C. difformi*s shown in the tree topology [17]. Thus, the ITS-2 sequence information is considered to contribute largely to the resolution of the basal phylogeny within the *Reticulata* group in the tree based on the present combined data set (Fig. 1).

### Paralogs of *rbcS* in the *Reticulata* group

**Fig. 2:**
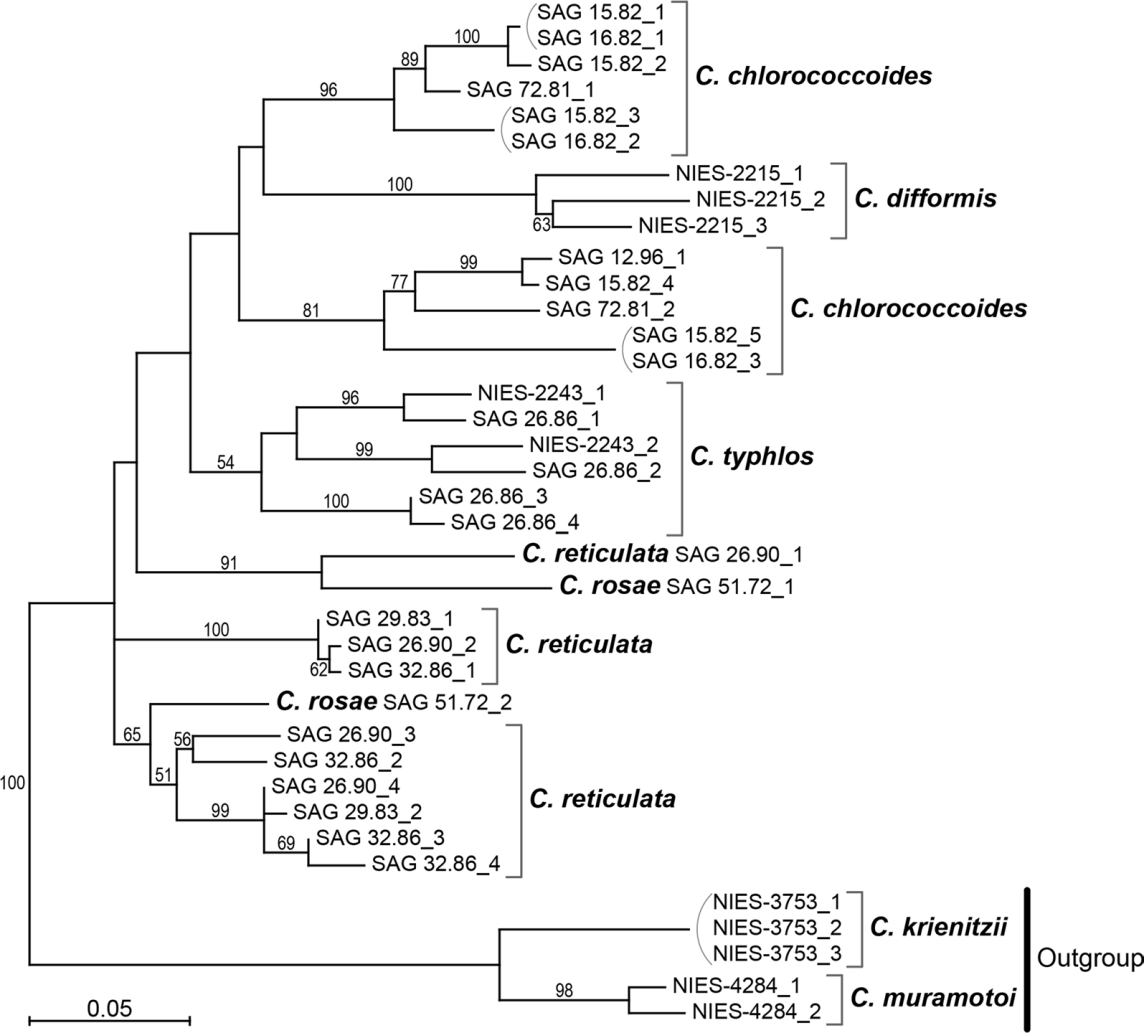
Phylogenetic analysis of *rbcS* sequences from the *Reticulata* group. The tree was constructed based on maximum likelihood method using 307 base pairs of coding regions of *rbcS* (Table 1). Numbers at the branches indicate bootstrap values (50% or more). For details, see Methods of the main text

Multiple *rbcS* sequences were detected in the de novo transcriptome assembly or cloned RT-PCR-products of all strains of the *Reticulata* group (Additional file 2: Table S1). In order to resolve the diversity and phylogeny of the *rbcS* paralogs in the *Reticulata* group, phylogenetic analyses were carried out. The *rbcS* phylogenetic tree did not resolve basal relationships of the genes in the *Reticulata* group (Fig. 2). However, six or three homologs from *C*. *typhlos* (two strains) or *C*. *difformis* (one strain), respectively, constituted a monophyletic group (Fig. 2). Three paralogs from a single strain (SAG 15.82) of *C. chlorococcoides* belonged to a clade composed of only strains of the same species. Six of 10 *C. reticulata rbcS* sequences formed a clade which included three paralogs from a single strain (SAG 32.86). Therefore, the *rbcS* genes might have been duplicated after the origin of each of the four *Chloromonas* species. Alternatively, gene conversion [20] could be considered to explain the apparent monophyly of the paralogs from the same species within the *Reticulata* group. Interestingly, two paralogs of *C. rosae* (one strain) were separated from each other, and each was sister to *C. reticulata* homolog(s) (Fig. 2). Because of presence of multiple *rbcS* paralogs in all five species and the discrepancy in the phylogeny of *C. rosae* and *C. reticulata* between the *rbcS* phylogeny and the previous species trees [16, 17], *rbcS* sequences were not used in the present study for phylogeny of species within the *Reticulata* group (Fig. 1).

### Hydrophobicity of RBCS helices

**Fig. 3:**
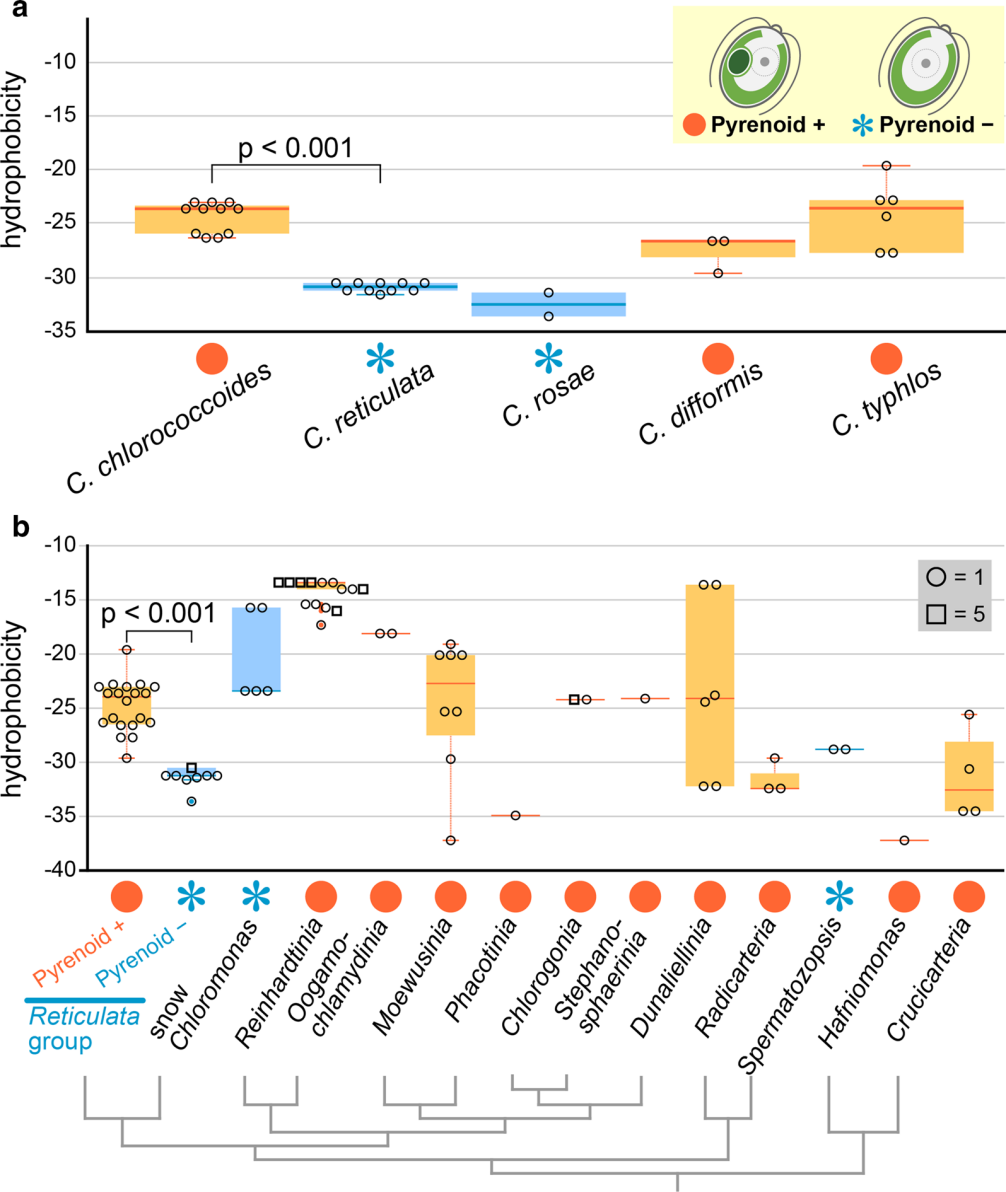
Box-whisker/bee swarm plots comparing the distribution of hydrophobicity of helices A and B from RBCS homologs of the Chlamydomonadales (Table 1). Significant difference (p < 0.001) is based on Brunner–Munzel test. **a** Comparison among five species of the *Reticulata* group of the genus *Chloromonas*. **b** Comparison among three groups of *Chloromonadinia* (the most left three), and 12 other lineages (11 strongly supported primary clades and *Spermatozopsis* [14]) within the Chlamydomonadales (Table 1), showing phylogeny based on Nakada et al. [14], Lemieux et al. [24] and Matsuzaki et al. [22, 27]

Table 1 shows the calculated hydrophobicity of RBCS helices A and B from the chlamydomonadalean species examined in this study. Figure 3 compares the hydrophobicity between pyrenoid-containing and -lacking species of Chlamydomonadales.

**Table 1:** List of species/strains of 12 lineages of the Chlamydomonadales [14] used for comparison of hydrophobicity scales of RBCS helices (Fig. 3)

Lineage or strongly supported primary clade [14]	Species	Strain	Sequence^a^	Source of *rbcS*	Accession number of *rbcS*	Hydrophobicity^b^
*Chloromonadinia* (*Reticulata* group with pyrenoid)	*Chloromonas chlorococcoides*	SAG 15.82	5	mRNA^c^	LC547865	− 23.6
					LC547866	− 23.6
					LC547867	− 23.0
					LC547868	− 25.9
					LC547869	− 26.3
		SAG 12.96	1	mRNA^c^	LC547870	− 25.9
		SAG 16.82	3	mRNA^c^	LC547871	− 23.6
					LC547872	− 23.0
					LC547873	− 26.3
		SAG 72.81	2	mRNA^c^	LC547874	− 23.6
					LC547875	− 23.0
	*Chloromonas difformis*	NIES-2215	3	mRNA^c^	LC547876	− 26.6
					LC547877	− 29.6
					LC547878	− 26.6
	*Chloromonas typhlos*	NIES-2243	2	mRNA^c^	LC547879	− 22.8
					LC547880	− 24.3
		SAG 26.86	4	mRNA^c^	LC547881	− 22.8
					LC547882	− 19.6
					LC547883	− 27.7
					LC547884	− 27.7
*Chloromonadinia* (*Reticulata* group without pyrenoid)	*Chloromonas reticulata*	SAG 29.83	2	mRNA^c^	LC547885	− 30.5
					LC547886	− 31.2
		SAG 26.90	4	mRNA^c^	LC547887	− 31.6
					LC547888	− 30.5
					LC547889	− 30.5
					LC547890	− 31.2
		SAG 32.86	4	mRNA^c^	LC547891	− 30.5
					LC547892	− 30.5
					LC547893	− 31.2
					LC547894	− 31.2
	*Chloromonas rosae*	SAG 51.72	2	mRNA^c^	LC547895	− 31.4
					LC547896	− 33.6
*Chloromonadinia* (snow *Chloromonas*)	*Chloromonas krienitzii*	NIES-3753	3	mRNA^c^	ICPW01000006	− 23.4
					ICPW01000007	− 23.4
					ICPW01000008	− 23.4
	*Chloromonas muramotoi*	NIES-4284	2	mRNA^c^	ICPX01000119	− 15.7
					ICPX01000120	− 15.7
*Crucicarteria*	*Carteria crucifera*	SAG 8-7a	1	mRNA^d^	VIAU scaffold 2059943	− 25.6
	*Carteria cerasiformis*	NIES-424	2	mRNA^c^	ICQZ01000174	− 34.5
					ICQZ01001652	− 30.6
		NIES-425	1	mRNA^c^	ICRA01000128	− 34.5
*Chlorogonia*	*Haematococcus lacustris*	NIES-144	4	gDNA	BLLF01000007	− 24.2
					BLLF01000015	− 24.2
					BLLF01001246	− 24.2
					BLLF01002948	− 24.2
		CCAC 0055	2	mRNA^d^	AGIO scaffold 2001898	− 24.2
					ODXI scaffold 2002606	− 24.2
*Dunaliellinia*	*Dunaliella parva*		1	mRNA	HQ315783	− 23.8
	*Dunaliella salina*		1	mRNA	AY739272	− 24.4
	*Dunaliella tertiolecta*		1	gDNA	AY530155	− 32.2
		CCMP 364	1	mRNA^d^	ZDIZ scaffold 2000214	− 32.2
	*Asteromonas gracilis*	CCAC 0049	2	mRNA^d^	MNPL scaffold 2000234	− 13.6
					NTLE scaffold 2002920	− 13.6
*Hafniomonas*	*Hafniomonas reticulata*	CCAC 0530/1	1	mRNA^d^	FXHG scaffold 2070561	− 37.2
*Moewusinia*	*Chlamydomonas moewusii*		1	mRNA	X13974	− 29.7
	*Chlamydomonas bilatus*	SAG 7.72	2	mRNA^d^	OVHR scaffold 3000834	− 25.3
					OVHR scaffold 3000835	− 25.3
	*Chlamydomonas eustigma*	NIES-2499	1	gDNA	BEGY01000097	− 37.2
	*Chlamydomonas* sp.	HS-5	1	mRNA	AU066498	− 20.1
	*Chlamydomonas* sp.	W80	3	mRNA	DC847488	− 20.1
					DC847494	− 20.1
					DC847626	− 19.1
*Oogamochlamydinia*	*Oogamochlamys gigantea*	SAG 44.91	1	mRNA^d^	XDLL scaffold 2047213	− 18.1
	*Lobochlamys segnis*	SAG 50.84	1	mRNA^d^	OFUE scaffold 2045013	− 18.1
*Phacotinia*	*Phacotus lenticularis*	SAG 61–1	1	mRNA^d^	ZIVZ scaffold 2002271	− 34.9
*Radicarteria*	*Carteria obtusa*	SAG 39.84	3	mRNA^d^	RUIF scaffold 2001762	− 29.6
					RUIF scaffold 2001763	− 32.4
					RUIF scaffold 2001764	− 32.4
*Reinhardtinia*	*Chlamydomonas reinhardtii*	CC-503cw92mt+	2	mRNA	XM_001702354	− 13.4
					XM_001702357	− 13.4
	*Chlamydomonas asymmetrica*	NIES-2207	2	gDNA	BDDA01000282 (2 sequences)	− 13.4
						− 13.4
	*Chlamydomonas cribrum*	SAG 13.72	1	mRNA^d^	BCYF scaffold 2001609	− 14.0
	*Chlamydomonas debaryana*	NIES-2212	3	gDNA	BDDB01000073	− 14.0
					BDDB01000328	− 14.0
					BDDB01001769	− 14.0
	*Chlamydomonas globosa*^e^	SAG 7.73	1	mRNA	EC116339	− 13.4
	*Chlamydomonas sphaeroides*	NIES-2242	3	gDNA	BDDC01000297 (2 sequences)	− 14.0
						− 14.0
					BDDC01001831	− 13.4
	“*Chloromonas*” *oogama*	SAG 9.79	1	mRNA^d^	IHOI scaffold 2001609	− 13.4
	*Gonium pectorale*	CCAC 0085	2	mRNA^d^	KUJU scaffold 2000900	− 16.0
					KUJU scaffold 2000901	− 16.0
	*Eudorina elegans*	CCAC 0011	3	mRNA^d^	RNAT scaffold 2001381	− 16.0
					RNAT scaffold 2001382	− 16.0
					RNAT scaffold 2001383	− 16.0
	*Heterochlamydomonas inaequalis*	SAG 4.75	1	mRNA^d^	IRYH scaffold 2037065	− 17.3
	*Lobomonas francei*	SAG 45–1	3	mRNA^d^	JKKI scaffold 2001057	− 15.4
					JKKI scaffold 2001059	− 15.4
					JKKI scaffold 2001061	− 15.7
	*Neochlorosarcina* sp.	CCAC 0208	1	mRNA^d^	USIX scaffold 2005162	− 14.0
	*Volvox aureus*	CCAC 1028	3	mRNA^d^	JWGT scaffold 2000603	− 13.4
					JWGT scaffold 2000604	− 13.4
					JWGT scaffold 2000606	− 13.4
		CCAC 2242	4	mRNA^d^	WRSL scaffold 2000117	− 13.4
					WRSL scaffold 2000118	− 13.4
					WRSL scaffold 2000119	− 13.4
					WRSL scaffold 2000120	− 13.4
	*Volvox carteri*		3	gDNA	AY205158 (3 sequences)	− 13.4
						− 13.4
						− 13.4
	*Volvox globator*	CCAC 2662	5	mRNA^d^	ASPU scaffold 2003346	− 13.4
					ASPU scaffold 2003347	− 13.4
					ASPU scaffold 2003348	− 13.4
					ASPU scaffold 2003349	− 13.4
					ASPU scaffold 2003350	− 13.4
*Stephanosphaerinia*	*Chlorococcum microstigmatum*^f^	SAG 11–43	1	mRNA^d^	QRTH scaffold 2038871	− 24.1
*Spermatozopsis*^g^	*Spermatozopsis similis*	CCAC 0013	2	mRNA^d^	ENAU scaffold 2000800	− 28.8
					ENAU scaffold 2000801	− 28.8

^a^Numbers of RBCS paralogs used for comparison of hydrophobicity scales of helices A and B (Fig. 3)

^b^Hydrophobicity of RBCS helices A and B. For details, see Methods

^c^For details, see Additional file 3: Table S2

^d^One thousand plant transcriptome data (https://db.cngb.org/onekp/) [48]

^e^Formerly identified as *Chlamydomonas incerta* [49]

^f^Formerly identified as *Chloromonas perforata* [50]

^g^Not identified as strongly supported primary clade because of its uncertain phylogenetic position [14]

Within the *Reticulata* group, for all 20 RBCS sequences, the hydrophobicity of the RBCS helices from the pyrenoid-containing species was higher (− 19.6 to − 29.6) than those of all 12 RBCS sequences from pyrenoid-lacking species (− 30.5 to − 33.6) (Fig. 3) (Table 1). The Brunner–Munzel test detected a significant difference (p < 0.001) between the pyrenoid-containing and -lacking species within the *Reticulata* group (Fig. 3). These results support previous studies that suggested that the interaction between pyrenoid-component proteins contributes to pyrenoid formation [8–12].

To investigate the correlation between the presence/absence of pyrenoids and the hydrophobicity of the RBCS helices among various lineages within Chlamydomonadales, the hydrophobicity of the helices was examined in 12 other lineages in the following order: snow *Chloromonas* species in the strongly supported primary clade *Chloromonadinia* [14], followed by 10 other strongly supported primary clades: (*Reinhardtinia*, *Oogamochlamydinia*, *Moewusinia*, *Phacotinia*, *Chlorogonia*, *Stephanosphaerinia*, *Dunaliellinia*, *Radicarteria*, *Hafniomonas*, and *Crucicarteria*) and *Spermatozopsis* [14] (Table 1). Both snow *Chloromonas* species lack pyrenoids [21, 22]. *Spermatozopsis* lacks pyrenoids [23]; its phylogenetic position is uncertain [14, 24] although it may be sister to *Radicarteria* [14]. *Hafniomonas* and *Crucicarteria* are positioned near the base of Chlamydomonadales and have a pyrenoid [23–25]. Species from other strongly primary clades examined in the present study (Table 1) possess pyrenoids [22, 23].

Comparing the hydrophobicity of the RBCS helices from various lineages within Chlamydomonadales showed that the hydrophobicity of the RBCS helices does not necessarily correspond to the presence or absence of a pyrenoid (Fig. 3b). The RBCS helices of pyrenoid-lacking snow *Chloromonas* species had relatively high hydrophobicities, whereas the values for *Phacotinia*, *Radicarteria*, *Hafniomonas* and *Crucicarteria* with pyrenoids in the chloroplast were low (Fig. 3b). Very recently, other protein factors were reported to contribute to pyrenoid formation in the *Chlamydomonas reinhardtii* chloroplast [8, 9, 11, 12]. Consequently, the hydrophobicity of the RBCS helices is not the only factor that controls pyrenoid formation. All snow species of *Chloromonas* lack pyrenoids and constitute a relatively large, pyrenoid-lacking lineage, the subclade 2 of clade A [26] or snow algae clade (SA clade) [21, 27], which is phylogenetically separated from the *Reticulata* group of *Chloromonas*. Thus, the common ancestral species of the extant snow *Chloromonas* species might have lacked pyrenoids in the chloroplast and diverged in the distant past; many changes in pyrenoid formation factors would have accumulated independently from the *Reticulata* group after divergence. Therefore, it is difficult to discuss pyrenoid presence/absence only in terms of the RBCS protein among the lineages within Chlamydomonadales. However, the evolutionary loss of pyrenoids within the *Reticulata* group is recognized as recent (Fig. 1), and the hydrophobicity of the RBCS helices differed significantly between pyrenoid-containing and -lacking species (Fig. 3). Therefore, during the initial stage of pyrenoid loss in the *Reticulata* group, changes in the hydrophobicity of the RBCS helices might have directly caused the disappearance of pyrenoids from the chloroplast.

### Comparison of sister species with and without pyrenoids

**Fig. 4:**
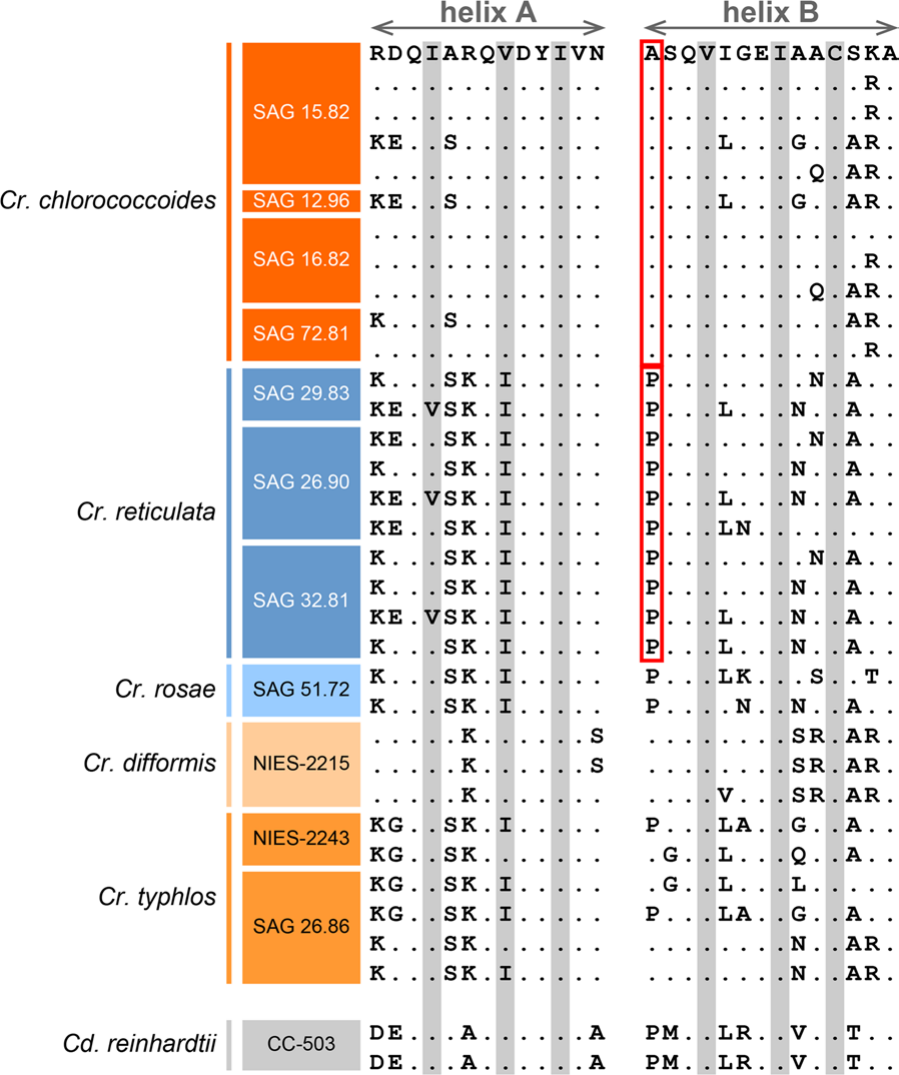
Amino acid alignment of helices A and B of RBCS homologs from the *Reticulata* group and *Chlamydomonas reinhardtii* (Table 1). Dot means that amino acid in the position is the same as that in the top sequence. Eliminated amino acids by less than 15% exposure ratio are grayed-out. The first position of helix B was alanine in all of the RBCS sequences from *Chloromonas chlorococcoides*, while proline in that from *C. reticulata* (surrounded by the red solid-line frames)

As discussed above, various molecular factors can be considered regarding the accumulation of Rubisco proteins to form pyrenoids [8–12]. Thus, comparison between closely related species with and without pyrenoids should be helpful to resolve critical factor causing pyrenoid loss/gain during speciation between these two species. Among sister species of the *Reticulata* group, *Chloromona*s *chlorococcoides* has and *C*. *reticulata* does not have pyrenoids (Fig. 1). To investigate differences in the amino acid sequences of the RBCS helices that may cause the difference in pyrenoid formation between these two species, the RBCS helices from these two species were compared: 11 sequences from four strains of *C*. *chlorococcoides* and 10 sequences from three strains of *C*. *reticulata* (Fig. 4). Within the 21 amino acid positions of helices A and B that were used to calculate hydrophobicity, one position differed markedly in amino acid hydrophobicity between these two species: the first position of helix B, corresponding to the 131 amino acid position in RBCS from *Chlamydomonas reinhardtii* [28]. The amino acid at this position in all of the *C*. *chlorococcoides* RBCS sequences was alanine (amino acid hydrophobicity = 1.8 [29]), while that in all of the *C*. *reticulata* RBCS sequences was proline (amino acid hydrophobicity =  − 1.6 [29]) (Fig. 4). Therefore, a mutation of this codon (− 3.4 difference in amino acid hydrophobicity) might have significantly contributed to the loss of pyrenoids during the divergence of the ancestor of the pyrenoid-lacking species *C*. *reticulata* from a common ancestral species that may have possessed pyrenoids. However, we consider that the loss of pyrenoids may be based on the total hydrophobicity of 21 amino acids of helices A and B of RBCS within the *Reticulata* group (Fig. 3a). Thus, hydrophobicity of the other 20 amino acid positions may also contributes to presence or absence of pyrenoids in the *Reticulata* group. Although the pyrenoid-containing species *C. typhlos* has proline in the 131 amino acid position of two of six RBCS proteins (Fig. 4), total amino acid hydrophobicity is relatively high (Fig. 3a).

## Discussion

In the present paper, we resolved possible correlation between the hydrophobicity of RBCS helices and presence/absence of pyrenoids in the *Reticulata* group of *Chloromonas*. This is possibly due to the unique fact that the *Reticulat*a group shows presence and absence of pyrenoids within closely related species or even between sister species (Figs. 1 and 3). We also found that the hydrophobicity of the RBCS helices does not necessarily correspond to the presence or absence of a pyrenoid among the large lineages (strongly supported primary clades [14]) within Chlamydomonadales (Fig. 3b). It is thus clearly difficult to resolve the correlation between the RBCS amino acid sequences and presence/absence of pyrenoids across major lineages of chlorophytes and streptophytes [13].

The environmental conditions play an important role in pyrenoid presence/absence in some species (e.g. *Volvulina steinii* [30]). However, the *Reticulata* group of *Chloromonas* does not show variability in presence or absence of pyrenoids within a species when cultured under usual light/dark conditions [6, 16–18]. Thus, presence or absence of the pyrenoids in the *Reticulata* group is not directly affected by cultural or environmental conditions, but it is genetically determined. The present study clearly demonstrated part of such genetic differences in *rbcS* genes between pyrenoid-containing and -lacking species.

## Conclusion

Recent extensive studies demonstrated that various molecular factors are possible to contribute to interaction between Rubisco and other proteins to form pyrenoids [8–12]. Loss of pyrenoids might have occurred many times in the distant past independently during the evolution of photosynthetic eukaryotes. Thus, it seems difficult to discuss the critical factor that might have directly caused the initial evolution of pyrenoid loss. Based on the use of the *Reticulata* group of *Chloromonas*, however, we here suggested that the hydrophobicity of the helices A and B of RBCS is a possible factor that might have caused the initial loss of pyrenoid during speciation between the pyrenoid-containing and -lacking species (Fig. 3a). Although the decrease in such hydrophobicity may be the major factor for evolutionary loss of pyrenoids in the *Reticulata* group, presence or absence of the EPYC-1-like protein is totally unknown in this group. RBCL amino acid substitutions may be related to the presence or absence of pyrenoids in *Chloromonas* [15]. Transformation protocols have not been established in *Chloromonas*. Further molecular genetic studies are needed to resolve actual molecular bases for evolutionary loss of pyrenoids in the *Reticulata* group.

## Methods

### Phylogenetic analyses of 11 *Chloromonas* strains of the *Reticulata* group

Molecular phylogenetic analyses were conducted based on Makino et al. [17] with additional sequences of nuclear internal transcribed spacer 2 (ITS-2) (Additional file 2: Table S1), using MrBayes 3.2.7 [31] for BI, RAxML-NG 0.9 [32] for ML method, and PAUP* 4.0b10 [33] for MP and NJ analyses. The combined 7109-bp data matrix for nuclear 18S and 28S ribosomal DNA, ATP synthase β-subunit (*atpB*), and P700 chlorophyll *a*-apoprotein A2 (*psaB*) and *psaA* genes, and ITS-2 sequences from the 14 operational taxonomic units (11 *Chloromonas* strains of the *Reticulata* group and three outgroup species) was analyzed and available from TreeBASE (https://www.treebase.org/treebase-web/home.html; study ID: S26516). For concatenating the data matrices, our previous studies showed that robust discrepancies in phylogenetic relationships within the *Chloromonadinia* clade were not detected among 18S rDNA, *atpB* and *psaB*-based trees [22, 27]. We also here confirmed that there are no robust discrepancies (supported > 60% bootstrap values) among 28S rDNA, *psaA* and ITS-2-based trees in the *Reticulata* group (Additional file 1: Fig. S1). The outgroup species were selected according to the previous phylogenetic analyses [16, 17, 27]. The appropriate substitution models for partitioned analyses in BI and ML method were selected by the Bayesian information criterion in Modeltest-NG v0.1.6 [34] with “-T mrbayes” option. The applied substitution models were as follows: K80+I for 18S ribosomal DNA, K80+G4 for 28S ribosomal DNA and ITS-2, HKY+I for the 1st codon position of *atpB*, *psaA* and *psaB*, F81+I+G4 for the 2nd codon position of *atpB*, *psaA* and *psaB*, and GTR+G4 for the 3rd codon position of *atpB*, *psaA* and *psaB*. BI was performed as in the previous study [17] with 1,000,000 generations of Markov chain Monte Carlo iterations and discarding the first 25% as burn-in. In each analysis, the average standard deviation of split frequencies was below 0.01, indicating convergence. For ML analysis, 10 randomized parsimony starting trees were generated. MP analysis was carried out based on random additions of 10 replicates from a heuristic search using the tree-bisection-reconnection branch-swapping algorithm. For NJ analysis, GTR+I+G model was selected by the Bayesian information criterion in jModelTest 2.1 [35]. Bootstrap values [19] based on 1000 replications were calculated in ML, MP and NJ analyses.

### Cultures

Eleven strains of five species in the *Reticulata* group [17] were obtained from the Culture Collection of Algae at Göttingen University (SAG; https://www.uni-goettingen.de/en/www.uni-goettingen.de/de/184982.html) and the Microbial Culture Collection at the National Institute for Environmental Studies (NIES; https://mcc.nies.go.jp/index_en.html): *Chloromonas reticulata* strains SAG 29.83, SAG 26.90, and SAG 32.86; *C*. *rosae* strain SAG 51.72; *C*. *chlorococcoides* strains SAG 15.82, SAG 12.96, SAG 16.82, and SAG 72.81; *C*. *typhlos* strains NIES-2243 and SAG 26.86; and *C*. *difformis* strain NIES-2215. Two strains of *Crucicarteria* (a strongly supported primary clade in the Chlamydomonadales [14]) were also obtained from NIES: *Carteria cerasiformis* strains NIES-424 and NIES-425. The cells were cultured in screw-cap tubes (18 × 150 mm) containing about 10 mL of AF-6 medium [36, 37] at 20 °C under 110–150 μmol photons m^−2^ s^−1^ light from fluorescent lamps with a 14-h light:10-h dark photoperiod. In addition, the snow algal species *Chloromonas krienitzii* strain NIES-3753 and *C*. *muramotoi* strain NIES-4284 were obtained from NIES and grown at 5 °C, as described previously [20].

### RNA extraction, library construction and sequencing

Total RNA from 11 *Chloromonas* strains of the *Reticulata* group and two snow species of *Chloromonas* (Additional file 3: Table S2) was isolated with TRIzol Reagent (Thermo Fischer Scientific, Carlsbad, CA, USA), as described by Featherston et al. [38] using cultures grown during the light photoperiod. The RNA was then treated using a TURBO DNA-*free* Kit (Thermo Fischer Scientific) to exclude genome DNA contamination and measured using an Agilent 4200 TapeStation (Agilent Technologies, Santa Clara, CA, USA). Ribosomal RNA was removed with a NEBNext Poly(A) mRNA Magnetic Isolation Module (New England Biolabs, Beverly, MA, USA), and sequencing libraries were prepared with the NEBNext Ultra Directional RNA Library Prep Kit for Illumina (New England Biolabs). The cDNA library was assessed using an Agilent 4200 TapeStation and NEBNext Library Quant Kit for Illumina (New England Biolabs). After normalization, paired-end sequencing (250 bp × 2) was performed using an Illumina MiSeq (Illumina, San Diego, CA, USA) with a MiSeq Reagent Kit v2 (500 cycles) (Illumina).

For the *Carteria cerasiformis* strains (Additional file 3: Table S2), total RNA was extracted from freeze-stocked cells using an RNeasy Mini Kit (QIAGEN, Venlo, the Netherlands) according to the manufacturer’s instructions. To synthesize the library, 1 μg of total RNA was treated as follows. The ribosomal RNA was removed using a Ribo-Zero rRNA Removal Kit (Plant Leaf) (Illumina) as per the manufacturer’s protocol. Sequencing libraries were prepared using the NEBNext mRNA Library Prep Kit for Illumina (New England Biolabs) with the following modifications. First-strand synthesis was performed without fragmenting the mRNA. After second-strand synthesis, double-stranded cDNA was fragmented to an average length of 500 bp using a Covaris S2 sonication system (Covaris, Woburn, CA, USA). Paired-end sequencing (300 bp × 2) was then conducted using an Illumina MiSeq with a MiSeq Reagent Kit v3 (600 cycles).

### De novo assembly

The number of paired-end reads sequenced using Illumina MiSeq was shown in Additional file 3: Table S2. For the *Chloromonas* species, sequence adapters and low-quality bases in the MiSeq reads were removed using Trimmomatic V0.38 [39]. Searching from both read ends, any base that had a quality value lower than 3 was removed. Sliding window trimming was performed with a 4-base window, and bases with quality scores under 15 were cut.

The processed reads of the *Chloromonas* strains were assembled de novo into contigs using Trinity V2.8.5 [40] (Additional file 3: Table S2). Using tblastn [41], the transcriptome libraries were searched for *rbcS* cDNA sequences, with the RBCS amino acid sequence of *Chlamydomonas reinhardtii* [28] serving as the search database. From the cDNA library, multiple contigs that contained the almost complete coding sequence (CDS) of *rbcS* were acquired (Additional file 2: Table S2).

The transcriptome reads of the two *Carteria cerasiformis* strains were filtered using CLC Genomics Workbench ver. 9.5 (QIAGEN) with following parameters: Phred quality score > 30; ambiguous nucleotides = 0; and removal of truncated reads less than 50 nucleotides in length. The filtered reads were assembled de novo using the CLC Genomics Workbench with the following parameters: automatic word size and bubble size; minimum contig length, 300 bp; correction of contig sequence by reads mapping; mismatch cost = 2; indel cost = 3; length fraction = 0.7; and similarity fraction = 0.9. Using the assembled contigs, the *rbcS* cDNA sequences were searched as described above (Additional file 3: Table S2).

### Cloning and sequencing

Because almost all strains of the *Reticulata* group possessed several *rbcS* paralogs (including partial CDS) in the de novo assembly, the cDNA sequences of the *rbcS* genes were verified by Sanger sequencing of the RT-PCR products. The cDNA was reverse-transcribed from the total RNA used for paired-end sequencing by Illumina MiSeq as described above, with Superscript III Reverse Transcriptase (Thermo Fischer Scientific). Approximately full-length paralog sequences of *rbcS* (covering CDS of helices A and B) were amplified by PCR with KOD FX Neo (Toyobo, Osaka, Japan) using newly designed primers based on our transcriptome data (Additional file 4: Table S3). The PCR products were cloned for sequencing using a Zero Blunt TOPO PCR Cloning Kit (pCR-Blunt II-TOPO Vector, Thermo Fischer Scientific) and TOPO TA Cloning Kit (pCR 4-TOPO Vector, Thermo Fischer Scientific). Then, 307 base pairs of the clones corresponding to positions 156–465 of the *Chlamydomonas reinhardtii rbcS* CDS (accession number XM_001702357) (with one amino acid deletion) were determined using a BigDye Terminator v3.1 Cycle Sequencing Kit (Applied Biosystems, Foster City, CA) and ABI Prism 3130 Genetic Analyzer (Applied Biosystems). To eliminate sequencing errors from the analysis, only identical sequences detected in at least two clones were used to calculate the hydrophobicity and phylogeny (Additional file 3: Table S2). The results by the Sanger sequencing partially conflicted with those of the assembled Illumina data, possibly due to the chimeric *rbcS* CDS resulting from the assembly of short similar sequences. Thus, only sequences of cloned *rbcS* were used in the present analyses (Additional file 3: Table S2).

### Phylogenetic analysis of *rbcS *paralogs from 11 *Chloromonas* strains of the *Reticulata* group

The 307 bp of *rbcS* cDNA from the 11 strains of the *Reticulata* group and the two snow species (outgroup) of the genus *Chloromonas* (Additional file 3: Table S2) were aligned with MAFFT V7.429 [42], and the phylogeny was analyzed with MEGA X [43]. ML analysis with 1000 bootstrap replications [19] was performed based on T92+G+I model selected by the Bayesian information criterion in MEGA X. The alignment is available at TreeBASE (https://www.treebase.org/treebase-web/home.html; study ID: S26516).

### Calculating the hydrophobicity of RBCS helices A and B

Following a previous study [10], 27 amino acids corresponding to positions 68–80 and 131–144 in the *Chlamydomonas reinhardtii* RBCS protein (including transit peptide; accession number XP_001702409) were regarded as helices A and B, respectively. The hydrophobicity of the two helices for each RBCS paralog was calculated as the sum of the hydrophobicity of the amino acid residues forming the helices, excluding embedded amino acid residues (i.e., not exposed to the surface). The amino acid positions corresponding to the embedded residues were investigated using the GetArea [44] (http://curie.utmb.edu/getarea.html); the three-dimensional (3D) structure *Chlamydomonas reinhardtii* RBCS [45] served as input. The exposure ratio was used to evaluate how “embedded” an amino acid was. The exposure ratio is the ratio of the side-chain surface area to the “random coil” value, i.e., the average solvent-accessible surface area of amino acid X in the tripeptide Gly-X-Gly in a set of 30 random conformations. Previous research regarded an amino acid with an exposure ratio below 15% as “embedded” [46]; hence, these residues were eliminated from the calculation of hydrophobicity of the RBCS helices. Consequently, we ignored six amino acids corresponding to positions 71, 75, 78, 134, 138, and 141 in the *Chlamydomonas reinhardtii* RBCS protein (accession number XP_001702409) within the 27 amino acids constituting the RBCS A and B helices (Additional file 5: Fig. S2). The Kyte–Doolittle hydrophobicity scale [29] was used for the hydrophobicity analysis.

### RBCS sequences of other Chlamydomonadales species

The RBCS sequences of other species in nine strongly supported primary clades (*i.e.*, *Chlorogonia*, *Dunaliellinia*, *Hafniomonas*, *Moewusinia*, *Oogamochlamydinia*, *Phacotinia*, *Radicarteria*, *Reinhardtinia*, and *Stephanosphaerinia*) and an “uncertain phylogenetic group” (*Spermatozopsis similis*) of Chlamydomonadales [14, 24] were extracted from published databases (Table 1). The sequences were manually checked, and those which were too short or possible cross-contamination were removed. The hydrophobicity of the RBCS helices was calculated as described above.

## Supplementary information

**Additional file 1: Fig. S1.** Bayesian phylogenetic trees of the *Reticulata* group of the genus *Chloromonas* based on 28S ribosomal DNA (a), *psaA* (b) and ITS-2 (c) sequences that constitute the combined data matrix for species phylogeny (Fig. 1).

**Additional file 2: Table S1.** Taxa/strains used for the phylogenetic analyses of the *Reticulata* group (Fig. 1) and DDBJ/ENA/GenBank accession numbers.

**Additional file 3: Table S2.** Summary of de novo transcriptome of *rbcS* cDNA in 11 strains of the *Reticulata* group, two snow species of the genus *Chloromonas* (*Cr.*), and two strains of *Carteria* (*Ca.*).

**Additional file 4: Table S3.** Newly designed primers for *rbcS* of the *Reticulata* group in *Chloromonas*.

**Additional file 5: Fig. S2.** 3D structure of RBCS showing the exposed and embedded amino acids of helices A and B.

## Data Availability

All relevant data are within the manuscript and its additional files. All sequence data have been deposited to NCBI/GenBank/DDBJ under the accessions as follows; DRA: DRR231178–DRR231190, DRR228661–DRR228664; transcriptome assembly: ICPZ01000001–ICPZ01001618, ICPY01000001–ICPY01011944, ICQA01000001–ICQA01014335, ICQG01000001–ICQG01001076, ICPU01000001–ICPU01018312, ICQD01000001–ICQD01019608, ICQC01000001–ICQC01000652, ICQE01000001–ICQE01006092, ICQF01000001–ICQF01001138, ICPV01000001–ICPV01021145, ICQB01000001–ICQB01000873, ICPW01000001–ICPW01001000, ICPX01000001–ICPX01001228, ICQZ01000001–ICQZ01028017, ICRA01000001–ICRA01031094; mRNA: LC547865–LC547896.

## Abbreviations

*atpB*: ATP synthase β-subunit
BI: Bayesian inference
CDS: Coding sequence
EPYC1: Essential pyrenoid component 1
ITS-2: Nuclear internal transcribed spacer 2
ML: Maximum likelihood
MP: Maximum parsimony
NJ: Neighbor-joining
*psaA*: P700 chlorophyll *a*-apoprotein A1
*psaB*: P700 chlorophyll *a*-apoprotein A2
RBCL: Rubisco large subunit
RBCS: Rubisco small subunit
RT-PCR: Reverse transcription-polymerase chain reaction
SA clade: Snow algae clade
