# Supplementary material for: The Rubisco small subunits in the green algal genus Chloromonas provide insights into evolutionary loss of the eukaryotic carbon-concentrating organelle, the pyrenoid
Source: BMC Ecol Evol. 2021 Jan 25;21:11. doi: 10.1186/s12862-020-01733-1 (PMC7853309; doi:10.1186/s12862-020-01733-1)
Supplement: Supplementary file 1 — Additional file 1: Fig. S1. Bayesian phylogenetic trees of the Reticulata group of the genus Chloromonas based on 28S ribosomal DNA (a), psaA (b) and ITS-2 (c) sequences that constitute the combined data matrix for species phylogeny (Fig. 1). [file 12862_2020_1733_MOESM1_ESM.docx]

**Fig. S1. Bayesian phylogenetic trees of the *Reticulata* group of the genus *Chloromonas* based on 28S ribosomal DNA (a), *psaA* (b) and ITS-2 (c) sequences that constitute the combined data matrix for species phylogeny (Fig. 1).**

Numbers shown in top left, top right, bottom left, and bottom right exhibit posterior probabilities (0.95 or more) from Bayesian inference (BI) and bootstrap values (50% or more) from maximum likelihood (ML), maximum parsimony, and neighbor-joining (NJ) analyses, respectively. The four phylogenetic methods were performed as described in the species phylogeny of the main text. In each tree, identical sequences were reduced to a single operational taxonomic unit. The appropriate substitution models for BI and ML analysis using MrBayes 3.2.7 [1] and RAxML-NG 0.9 [2], respectively, were selected by the Bayesian information criterion in Modeltest-NG v0.1.6 [3] with “-T mrbayes” option. The applied models were HKY+G4 for 28S ribosomal DNA, K80+I, JC+I, and HKY+G4 for 1st, 2nd, and 3rd codon positions of *psaA*, respectively, and HKY+G4 for ITS-2. The appropriate substitution models for NJ analysis using PAUP* 4.0b10 [4] were selected by the Bayesian information criterion in jModelTest 2.1 [5]. The selected models were TrN+G for 28S ribosomal DNA, TIM1+I+G for *psaA*, and SYM+G for ITS-2.

**References**

1. Lonquist FM, Teslenko P, van der Mark DL, Ayres A, Darling S, Höhna B, et al. MrBayes 3.2: efficient Bayesian phylogenetic inference and model choice across a large model space. Syst Biol. 2012;61: 539–542. doi: 10.1093/sysbio/sys029.

2. Kozlov AM, Darriba D, Flouri T, Morel B, Stamatakis A. RAxML-NG: a fast, scalable and user-friendly tool for maximum likelihood phylogenetic inference. Bioinformatics. 2019;35: 4453–4455. doi: 10.1093/bioinformatics/btz305.

3. Darriba D, Posada D, Kozlov AM, Stamatakis A, Morel B, Flouri T. ModelTest-NG: a new and scalable tool for the selection of DNA and protein evolutionary models. Mol Biol Evol. 2020;37: 291–294. doi: 10.1093/molbev/msz189.

4. Swofford DL. PAUP*: phylogenetic Analysis Using Parsimony (* and other methods) [CD-ROM]. Version 4.0b10. Sinauer: Sunderland, MA. 2002.

5. Posada D. jModelTest: phylogenetic model averaging. Mol Biol Evol. 2008;25: 1253–1256. doi: 10.1093/molbev/msn083.
