# Supplementary material for: The Rubisco small subunits in the green algal genus Chloromonas provide insights into evolutionary loss of the eukaryotic carbon-concentrating organelle, the pyrenoid
Source: BMC Ecol Evol. 2021 Jan 25;21:11. doi: 10.1186/s12862-020-01733-1 (PMC7853309; doi:10.1186/s12862-020-01733-1)
Supplement: Supplementary file 2 — Additional file 2: Table S1. Taxa/strains used for the phylogenetic analyses of the Reticulata group (Fig. 1) and DDBJ/ENA/GenBank accession numbers. [file 12862_2020_1733_MOESM2_ESM.docx]

**Table S1. Taxa/strains used for the phylogenetic analyses of the *Reticulata* group (Fig 1) and DDBJ/ENA/GenBank accession numbers.**

|  |  | Accession number | | | | | |
| --- | --- | --- | --- | --- | --- | --- | --- |
| Taxon | Strain | SSU rDNA | LSU rDNA | *atpB* | *psaA* | *psaB* | ITS-2 |
| *Chloromonas chlorococcoides* | SAG 15.82 | AJ410449 | AB906359 | AB624580 | AB624583 | AB624595 | AB624570 |
|  | SAG 12.96 | AJ410451 | LC438810 | AB624581 | AB624584 | AB624596 | AB624571 |
|  | SAG 16.82 | AJ410450 | LC438809 | AB624582 | AB624585 | AB624597 | AB624572 |
|  | SAG 72.81 | U70785 | LC438806 | AB084309 | AB624586 | AB084343 | AB624573 |
| *Chloromonas difformis* | NIES-2215  (= CCAP 11/72) | AB701536 | LC438811 | LC438815 | LC438818 | LC438814 | FR865610 |
| *Chloromonas reticulata* | SAG 29.83  (= CCCryo 213-05, UTEX 1970) | U70791 | LC438808 | AB084312 | AB624587 | AB084346–7 | HQ404885 |
|  | SAG 26.90 | AB624563–4, AF517090 | LC438803 | AB084316 | AB624589 | AB084352–3 | AB624575 |
|  | SAG 32.86 | AB624561–2, AF517091 | LC438805 | AB084314 | AB624588 | AB084349 | AB624574 |
| *Chloromonas rosae* | SAG 51.72  (= UTEX 1337) | AB624565 | LC438804 | AB084315 | AB624590 | AB084350–1 | AB624576 |
| *Chloromonas typhlos* | NIES-2243 | AB701538 | LC438807 | LC438816 | LC438817 | LC438813 | LC439250 |
|  | SAG 26.86  (= UTEX 1969) | AB624566 | LC438802 | AB084307 | AB624591 | AB084341 | HQ404869 |
| Outgroup |  |  |  |  |  |  |  |
| *Chloromonas augustae* | SAG 5.73 | AJ410452 | LC360474 | AB504757 | AB624592 | AB504769 | AB624577 |
| *Chloromonas serbinowii* | SAG 11.82  (= UTEX 492) | AB624568–9, U70795 | LC360478 | AB084317 | AB624594 | AB084354 | AB624579 |
| *Gloeomonas anomalipyrenoides* | NIES-447 | AB504776 | LC437681 | AB084313 | AB624593 | AB084348 | AB624578 |

Abbreviations: *atpB*, ATP synthase beta subunit gene; ITS-2, internal transcribed spacer 2 of ribosomal DNA; LSU rDNA, the large subunit of ribosomal DNA; *psaA*, P700 chlorophyll *a* apoprotein A1 gene; *psaB*, P700 chlorophyll *a* apoprotein A2 gene; SSU rDNA, the small subunit of ribosomal DNA.
