# Supplementary material for: The Rubisco small subunits in the green algal genus Chloromonas provide insights into evolutionary loss of the eukaryotic carbon-concentrating organelle, the pyrenoid
Source: BMC Ecol Evol. 2021 Jan 25;21:11. doi: 10.1186/s12862-020-01733-1 (PMC7853309; doi:10.1186/s12862-020-01733-1)
Supplement: Supplementary file 3 — Additional file 3: Table S2. Summary of de novo transcriptome of rbcS cDNA in 11 strains of the Reticulata group, two snow species of the genus Chloromonas (Cr.), and two strains of Carteria (Ca.). [file 12862_2020_1733_MOESM3_ESM.docx]

**Table S2. Summary of *de novo* transcriptome of *rbcS* cDNA in 11 strains of the *Reticulata* group, two snow species of the genus *Chloromonas* (*Cr.*), and two strains of *Carteria* (*Ca.*).**

| Species | Strain | Total read^1^  [Accession no.] | Contig^2^  [Accession no.] | Contig with a full *rbcS* cds^3^ | Analyzed *rbcS* type^4^ |
| --- | --- | --- | --- | --- | --- |
| ***Reticulata* group** | | | | | |
| *Cr.* *chlorococcoides* | SAG 15.82 | 209204  [DRR231179] | 1618  [ICPZ01000001–ICPZ01001618] | 3 | 5 |
|  | SAG 12.96 | 220485  [DRR231178] | 11944  [ICPY01000001–ICPY01011944] | 8 | 1 |
|  | SAG 16.82 | 183503  [DRR231180] | 14335  [ICQA01000001–ICQA01014335] | 6 | 3 |
|  | SAG 72.81 | 129665  [DRR231181] | 1076  [ICQG01000001–ICQG01001076] | 2 | 2 |
| *Cr. difformis* | NIES-2215 | 219283  [DRR231186] | 18312  [ICPU01000001–ICPU01018312] | 4 | 3 |
| *Cr. reticulata* | SAG 29.83 | 247732  [DRR231183] | 19608  [ICQD01000001–ICQD01019608] | 6 | 2 |
|  | SAG 26.90 | 86226  [DRR231182] | 652  [ICQC01000001–ICQC01000652] | 5 | 4 |
|  | SAG 32.86 | 45406  [DRR231184] | 6092  [ICQE01000001–ICQE01006092] | 6 | 4 |
| *Cr. rosae* | SAG 51.72 | 136157  [DRR231185] | 1138  [ICQF01000001–ICQF01001138] | 4 | 2 |
| *Cr. typhlos* | NIES-2243 | 188643  [DRR231187] | 21145  [ICPV01000001–ICPV01021145] | 2 | 2 |
|  | SAG 26.86 | 112393  [DRR231188] | 873  [ICQB01000001–ICQB01000873] | 1 | 4 |
| **Snow species** | | | | | |
| *Cr. krienitzii* | NIES-3753 | 122120  [DRR231189] | 1000  [ICPW01000001–ICPW01001000] | 3^5^ | − |
| *Cr. muramotoi* | NIES-4284 | 101534  [DRR231190] | 1228  [ICPX01000001–ICPX01001228] | 2^5^ | − |
| ***Carteria*** | | | | | |
| *Ca. cerasiformis* | NIES-424 | 50718894  [DRR228661, DRR228663] | 28017  [ICQZ01000001–ICQZ01028017] | 2^5^ | − |
|  | NIES-425 | 41627514  [DRR228662, DRR228664] | 31094  [ICRA01000001–ICRA01031094] | 1^5^ | − |

^1^Number of the reads from Illumina MiSeq sequencing.

^2^Number of contigs from *de novo* assembly.

^3^Number of full length of *rbcS* coding sequence from *de novo* assembly.

^4^Number of different *rbcS* sequences obtained from Sanger-sequencing of cloned RT-PCR-products (for details, see Methods in the main text). These sequences were obtained because the *Reticulata* group exhibited possible chimeric *rbcS* paralogs in *de novo* assembly.

^5^Directly used for the present analyses (Table 1).
