# Supplementary material for: The Rubisco small subunits in the green algal genus Chloromonas provide insights into evolutionary loss of the eukaryotic carbon-concentrating organelle, the pyrenoid
Source: BMC Ecol Evol. 2021 Jan 25;21:11. doi: 10.1186/s12862-020-01733-1 (PMC7853309; doi:10.1186/s12862-020-01733-1)
Supplement: Supplementary file 4 — Additional file 4: Table S3. Newly designed primers for rbcS of the Reticulata group in Chloromonas. [file 12862_2020_1733_MOESM4_ESM.docx]

**Table S3. Newly designed primers for *rbcS* of the *Reticulata* group in *Chloromonas*.**

| **Designation** | **Positions^1^** | **Sequence (5'–3')** |
| --- | --- | --- |
| rbcS_F | 128–155 | CCAACCTSTTYAAGGTCTGGAAGGAGGC |
| rbcS_R^2^ | 491–466 | CARATCTGSACCTGRCGRATGTTGTC |

^1^Corresponding to the coding sequence of *Chlamydomonas reinhardtii* *rbcS*1 mRNA (XM_001702357).

^2^Reverse primer.
