# Supplementary material for: The Rubisco small subunits in the green algal genus Chloromonas provide insights into evolutionary loss of the eukaryotic carbon-concentrating organelle, the pyrenoid
Source: BMC Ecol Evol. 2021 Jan 25;21:11. doi: 10.1186/s12862-020-01733-1 (PMC7853309; doi:10.1186/s12862-020-01733-1)
Supplement: Supplementary file 5 — Additional file 5: Fig. S2. 3D structure of RBCS showing the exposed and embedded amino acids of helices A and B. [file 12862_2020_1733_MOESM5_ESM.docx]

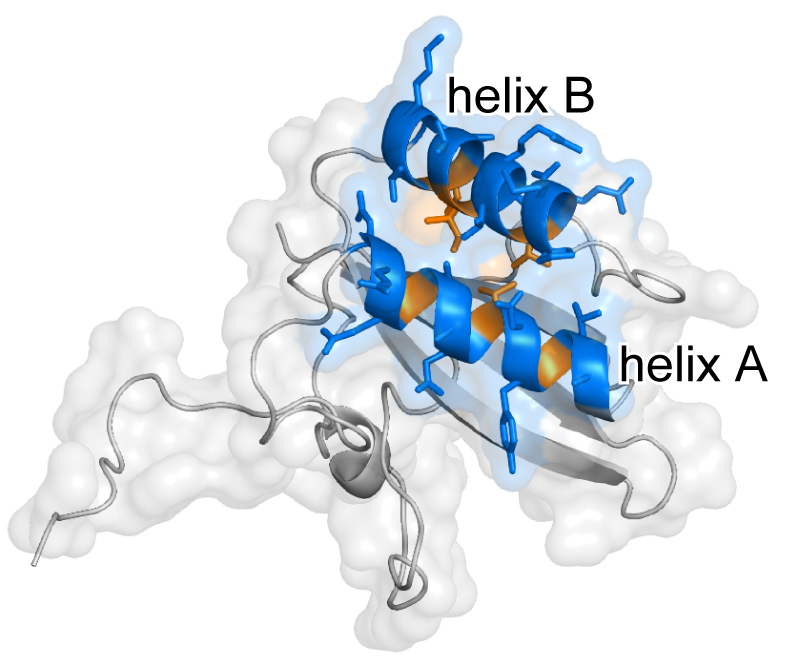


**Fig. S2. 3D structure of RBCS showing the exposed and embedded amino acids of helices A and B.**

Orange-colored amino acid sites in helices A and B were considered to be embedded. Only exposed amino acid sites in the helices (blue) were considered in the present hydrophobicity analysis of the RBCS helices. 3D structure was constructed based on *Chlamydomonas reinhardtii* RBCS 3D structure [1]. The 3D structure was visualized by PyMOL [2].

**References**

1. Taylor TC, Backlund A, Bjorhall K, Spreitzer RJ, Andersson I. First crystal structure of Rubisco from a green alga *Chlamydomonas reinhardtii*. J Biol Chem. 2001;276: 48159–48164. doi: 10.1074/jbc.M107765200.
2. The PyMOL Molecular Graphics System, Version 2.0 [software]. Schrödinger, LLC. 2017 [cited 2019 March 13]. Available from: https://pymol.org/2/#page-top.
